# Assessment of mercury emissions into the atmosphere from the combustion of hard coal in a home heating boiler

**DOI:** 10.1007/s11356-019-05432-3

**Published:** 2019-05-31

**Authors:** Tadeusz Dziok, Przemysław Grzywacz, Piotr Bochenek

**Affiliations:** 1 Faculty of Energy and Fuels, AGH University of Science and Technology, Al. A. Mickiewicza 30, 30-059 Krakow, Poland; 2 AGH Centre of Energy, AGH University of Science and Technology, ul. Czarnowiejska 36, 30-054 Krakow, Poland

**Keywords:** Hard coal, Home heating boiler, Mercury, Distribution, Emission

## Abstract

The purpose of the paper was to determine the factor of mercury emission into the atmosphere by households in Poland. Research for a home coal-fired boiler typical of Polish conditions was carried out, which was conducted throughout the heating season. On the basis of assessment of the quantity of coal burned and mercury content contained therein, as well as of the mercury content in bottom ash, chimney soot, boiler deposits and their quantities, annual mercury emissions and its factor of emission into the atmosphere were defined. It was defined that the mercury emission factor for the investigated case of a single-family house is at a level of 0.56 μg/MJ. It was shown that 41.4% of the mercury contained in coal burned in a home heating boiler is emitted into the atmosphere, 57.0% is adsorbed by chimney soot, 0.3% by boiler heater deposits and 1.3% passes into bottom ash. Annual mercury emissions into the atmosphere from the single-family house concerned was 79 mg. Mercury emissions can be significantly reduced by households by separating any overgrowths with pyrite from coal. The solution proposed would enable a reduction in annual mercury emissions into the atmosphere in Poland from the domestic user sector by 58.5% (0.351 Mg). The factor of emission of mercury into the atmosphere would be 0.23 μg/MJ.

## Introduction

Hard coal is one of the primary sources of the anthropogenic emission of mercury into the atmosphere (Pacyna et al. 2016; Pirrone et al. 2010), which is characterised by highly toxic properties (Li and Tse 2015). Therefore, efforts with a view to reducing its emissions are currently being made. An example of these efforts is the adoption in the European Union of emission limits for the power industry (BAT-LCP 2017), which come into effect in 2021. It is especially important in the case of countries such as Poland, for which hard coal is the primary energy carrier. The consumption of hard coal in Poland amounted to 74.2 million Mg in 2016, of which for electrical energy and heat generation in the power industry and industrial installations-43.8 million Mg, in industry-17.6 million Mg and in the municipal and domestic sector-12.8 million Mg (GUS 2017).

A special case is the use of hard coal by households. In contrast to power plants, home heating boilers are not equipped with a flue gas treatment system. They are also a source of so-called low emissions (GIOŚ 2017), which is the major cause of smog in Polish cities and villages. It should also be mentioned that part of the mercury emitted into the atmosphere is adsorbed on the surface of particulate matter (AEA Technology and NILU-Polska 2005; Chyc and Burzała 2012), which enhances the adverse effect of smog. A reduction in the low emissions can be secured by using other fuels, e.g. natural gas (Czerski et al. 2013; Czerski and Strugała 2014). However, on account of the lower costs of heating with hard coal by approx. 24% compared to natural gas, this method of heating will continue to remain predominant in Poland in the years to come (Stala-Szlugaj 2017).

In 2016, households in Poland emitted 0.6 Mg of mercury into the atmosphere (KOBiZE 2018). For determining the size of these emissions, a factor method is employed, i.e. coal consumption is multiplied by the emission factor. The emission factor is defined as the quantity of mercury emitted into the atmosphere in relation to the calorific value of coal burned. For households in Poland, it is presumed that the level of this factor is 2.0 μg/MJ. The uncertainty of an estimation of the size of mercury emissions from this source was 58% (KOBiZE 2018). The degree of uncertainty is affected inter alia by the diversified mercury content in Polish hard coal, which varies between 13 and 156 μg/kg (Wichliński et al. 2013), but also by the type of a heating boiler. The factor of emission of mercury for different types of boilers varies between 1.4 and 2.1 μg/MJ (Kubica 2017).

The purpose of the paper was to determine the factor of emission into the atmosphere by households in Poland. Research for a home coal-fired boiler typical of Polish conditions was carried out, which was conducted throughout the heating season. On the basis of assessment of the quantity of coal burned and mercury content contained therein, as well as of the mercury content in bottom ash, chimney soot, boiler deposits and their quantities, annual mercury emissions and its factor of emission into the atmosphere were defined. Furthermore, the efforts which enable the reduction of mercury emissions from the processes of combustion of hard coal in the domestic user sector in Poland were proposed.

## Experimental and analytical procedures

### Combustion of coal in a home heating boiler

**Fig. 1:**
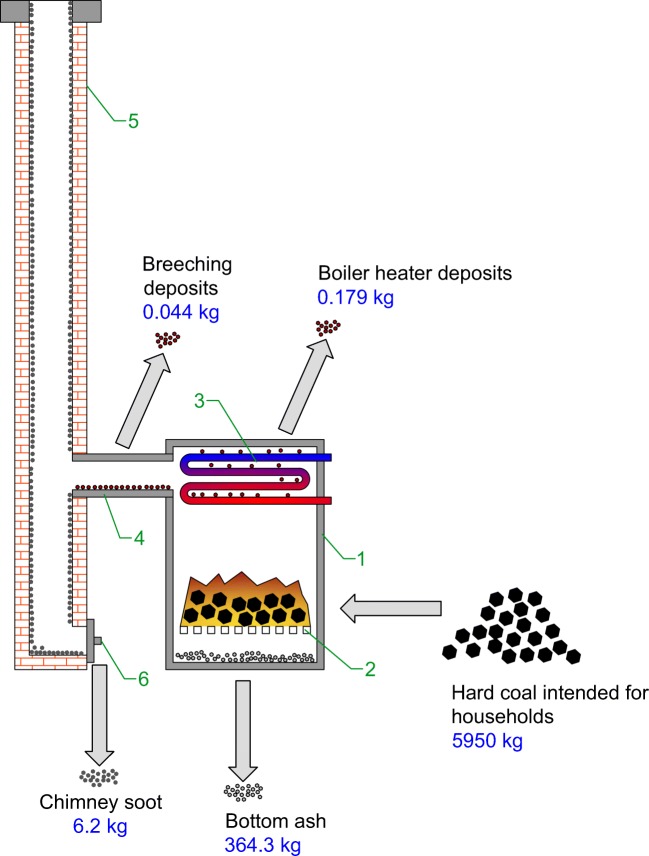
Diagram of the tested boiler with a mass of analysed fluxes for an entire heating season (1–home coal-fired boiler; 2–furnace with a grid; 3–heater; 4–breeching; 5–chimney; 6–cleanout opening)

The research was conducted for a coal-fired boiler heating a single-family house with a floor area of 200 m^2^. The research was conducted for an entire heating season. The boiler parameters are provided in Table 1. The pictorial diagram of the tested boiler with specifying streams analysed and their mass for an entire heating season is shown in Fig. 1. The research covered hard coal, bottom ash, deposits being accumulated in the boiler heater and flue pipes joining the boiler with the chimney (breeching), as well as soot being accumulated in the chimney.

**Table 1:** Technical data of the boiler

Detail	Value
Power [kW]	25
Efficiency [%]	≥ 78
Water temperature at the outlet [°C]	60
Year of manufacture	2003
Manufacturer	ZGM Zębiec
Boiler condition	Good

### Coal analysis

**Fig. 2:**
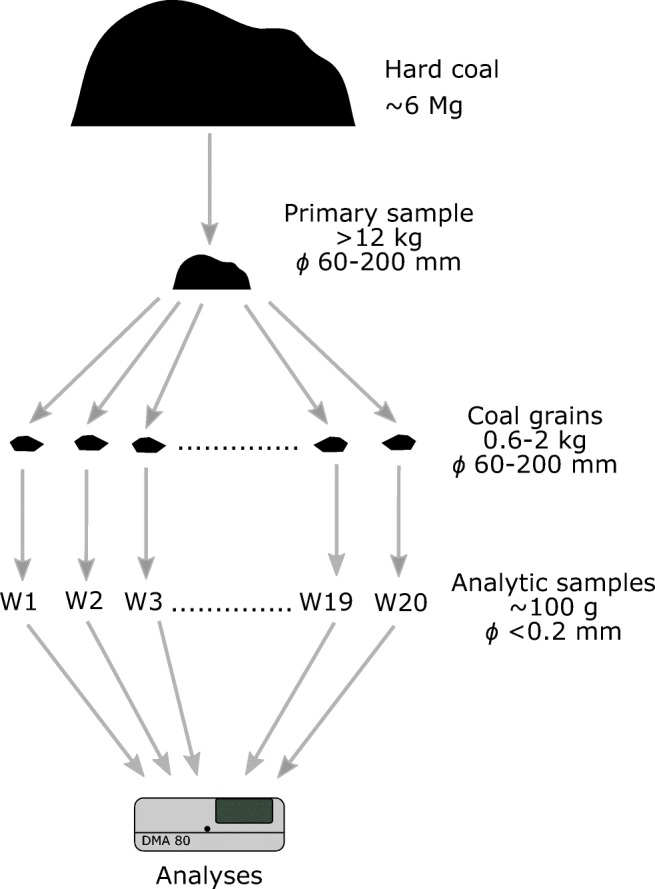
Coal sampling scheme

The analysed coal was bought from a coal distributor in a coal grain size of 60–200 mm. The coal was from KWK Piast, a Polish hard coal mine. For the heating season, 6 Mg of coal was bought. The scheme of sampling is shown in Fig. 2. From the coal lot, a representative sample with a mass of about 12 kg (the primary sample) was taken for analysis. The problem of variability of mercury content in hard coal is widely known (Wichliński et al. 2013). In order to measure the variability of occurrence of mercury, 20 coal grains were randomly taken from the primary sample (with a particle size distribution of 60–200 mm). Out of the grains, analytical samples (the samples were successively labelled from W1 to W20) with a particle size distribution below 0.2 mm were prepared, and mercury content was determined in them. The principal proximate and ultimate analyses were performed. The specifications of the analysed coal are provided in Table 2.

**Table 2:** Specifications of the analysed coal

Parameter	Proximate analysis				Ultimate analysis			
	M^ar^ [%]	A^ar^ [%]	V^daf^ [%]	LHV^ar^ [MJ/kg]	C^d^ [%]	H^d^ [%]	S_t_^d^ [%]	Hg^d^ [μg/kg]
Mean	14.0	4.6	36.90	23.487	74.8	3.46	0.96	38
Min. value	8.7	2.0	34.38	21.057	67.8	3.02	0.40	1
Max. value	18.8	12.0	38.44	25.729	78.6	3.91	3.37	261
Dispersion	10.1	10.0	4.06	4.672	10.8	0.89	2.97	260

### Bottom ash analysis

Samples of bottom ash were taken throughout the heating season from November to April. The ash was collected in plastic bags and closed tightly. In order to measure the variability of occurrence of mercury in the bottom ash, out of the ash collected, 20 analytical samples corresponding to a period of 1–2 week(s) were prepared (the samples were successively labelled from P1 to P20). It is widely known that mercury in coal co-occurs with sulphur (Diehl et al. 2004; Zheng et al. 2008), and in fly ash, its content is correlated with the quantity of unburned carbon (Burmistrz et al. 2016). Therefore, in the ash samples, the content of mercury, total sulphur and of carbon was determined (Table 3). Furthermore, in order to compare the bottom ash with the deposits being accumulated in the boiler and breeching heater, their basic composition was determined using the EDXRF technique (Table 4).

**Table 3:** Specifications of the bottom ash

Parameter	M^ar^ [%]	C^d^ [%]	S_t_^d^ [%]	Hg^d^ [μg/kg]
Mean	1.9	29.8	2.63	8
Min. value	1.1	21.6	2.24	3
Max. value	3.0	37.8	3.11	12
Difference	1.9	16.2	0.87	9

**Table 4:** Basic composition of the bottom ash and samples of deposits taken from the boiler

Analysed sample	Content of elements in oxide form [%]										
	SiO_2_	Al_2_O_3_	Fe_2_O_3_	CaO	MgO	K_2_O	P_2_O_5_	SO_3_	MnO	SrO	TiO_2_
Bottom ash	22.2	20.6	9.0	8.6	3.4	0.4	0.4	6.9	0.2	0.1	1.0
Heater deposit	13.8	13.0	7.0	5.8	1.7	2.1	0.4	27.4	0.1	0.1	0.4
Breeching deposit	25.3	13.3	3.3	8.2	1.9	1.8	0.5	18.0	0.1	0.1	0.4

### Boiler deposit analysis

After the end of the heating season, the boiler heater and breeching were cleaned, and the mass of the deposits obtained was determined. In the deposits, mercury content and the basic composition of ash were determined using the EDXRF technique (Table 4). It can be observed that the boiler deposits are characterised by a significantly higher sulphur content compared to the bottom ash.

### Chimney soot analysis

After the end of the heating season, a sample of soot was also taken when cleaning the chimney, and its mass was determined. The chimney was cleaned by a master chimney sweep according to the applicable provisions. In the soot, mercury content was determined.

### Methods and results of determining mercury content

For all the samples, mercury content was determined with the use of a DMA-80 mercury analyser. The device uses the atomic absorption spectrometry technique. A sample is subjected to thermal decomposition in an oxygen atmosphere. The resulting gases are steered to the catalyst, which removes the impurities which cause interference, and then, mercury is caught by a gold trap, the so-called amalgamator. During the following step of the analysis, mercury is discharged from the amalgamator and steered to a measuring tray. As a radiation source, a low-pressure mercury-discharge lamp was used with a wave length of 253.65 nm, and as a detector, a siliceous ultraviolet photodiode was used. The method was positively verified with the use of SRM 1632d and SRM 2685c standard reference materials. The results of validation for the repeatability of the method of determining mercury content for selected samples are provided in Table 5. In the case of coal and ash, the results for the samples for which mercury content was the highest, the lowest and close to the mean are provided. In the case of soot, a high value of standard deviation and uncertainty can be explained by the very high mercury content.

**Table 5:** The results of validation for the repeatability of the method of determining mercury content for selected samples

Material under analysis	Sample reference	Number of measurements	Mean [μg/kg]	Standard deviation [μg/kg]	Uncertainty^1)^ [μg/kg]	Relative uncertainty [%]
Coal	W9	3	33.1	2.2	± 2.5	8
	W11	4	213.9	25.0	± 25.0	16
	W20	3	1.0	0.02	± 0.02	2
Bottom ash	P1	3	11.7	0.5	± 0.5	5
	P8	3	6.8	0.7	± 0.8	12
	P13	3	3.2	0.2	± 0.2	6
Deposits/chimney soot	Heater deposit	3	1220	14	± 16	1
	Breeching deposit	3	1518	19	± 22	1
	Chimney soot	3	17,519	128	± 146	1

^a^Uncertainty estimated at a level of confidence of 0.95

## Results and discussion

### Mercury content in the analysed coal

**Fig. 3:**
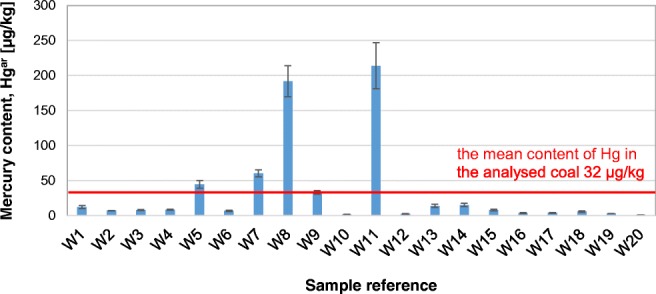
Comparison of mercury content in grains of the analysed coal (uncertainty shown as whiskers)

**Fig. 4:**
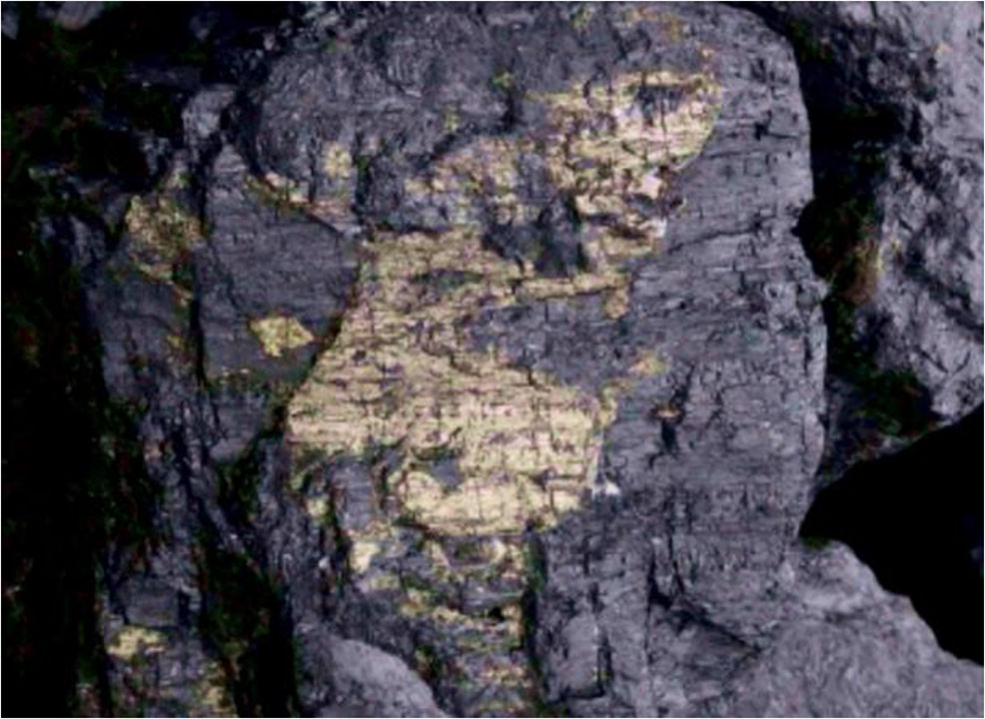
Pyrite overgrowth on grain of the analysed coal—sample W11

In Fig. 3, the dispersion of mercury content in the analysed coal is shown. Mercury content in individual grains varied dramatically from 1 to 214 μg/kg (as received basis). The mean mercury content in the coal analysed was 32 μg/kg. This is a relatively low content, but typical of coal intended for households, which is from 7 to 85 μg/kg with a mean of 42 μg/kg (Klojzy-Karczmarczyk and Mazurek 2013). The very high mercury content in samples W8 and W11 may be the result of pyrite overgrowths (Diehl et al. 2004), which was noticed previously at the stage of sample preparation (Fig. 4). These samples were also characterised by a high sulphur content, 2.16% for sample W8 and 2.76% for sample W11, respectively.

### Mercury content in the bottom ash

**Fig. 5:**
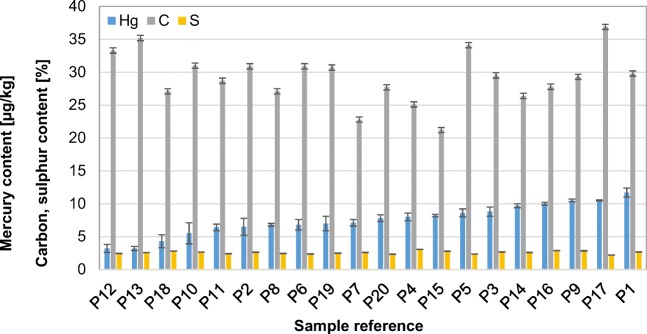
Comparison of mercury, carbon and sulphur content in the bottom ash from the boiler (uncertainty deviation shown as whiskers)

Mercury content in the bottom ash samples achieved a stable level from 3 to 12 μg/kg with a mean of 8 μg/kg—on a dry basis (Fig. 5). The ash samples were also characterised by a high sulphur content, from 2.24 to 3.11%, and a relatively high content of unburned carbon, from 21.6 to 27.8% (on a dry basis). The statistical analysis did not show any significant relationship between mercury content and the content of unburned carbon and total sulphur in the bottom ash. Interestingly, mercury content in the ash from the tested boiler was similar to that in slag from a coal power plant, which varies between 8 and 10 μg/kg. In light of the accessible data, the major factor determining mercury content in the bottom ash may be its high volatility (Clarke and Sloss 1992).

### Mercury content in deposit samples taken from the boiler and in chimney soot

In comparison to the coal and bottom ash, the samples of deposits taken from the boiler were characterised by a very high mercury content amounting to 1220 (heater deposits) and 1518 μg/kg (breeching deposits), respectively. Out of all of the samples analysed, it was the chimney soot that was characterised by the highest mercury content—17,514 μg/kg. The high mercury content in soot could be explained by both the very good adsorptive properties of soot and the long contact time of soot with the mercury contained in flue gas (up to a few months). By settling on the chimney walls, soot formed an effective layer of an adsorbent. Furthermore, it was found that other ecotoxic elements are also adsorbed in large quantities on the surface of chimney soot, e.g. As, Pb, Cu, Zn, Ni, Cr (Chyc and Burzała 2012), which are commonly present in hard coal (Makowska et al. 2019).

On the one hand, adsorbing mercury by chimney soot is advantageous, because it reduces emissions into the atmosphere, but on the other, this results in it having harmful properties to health. Therefore, in the case of contact with soot, one must abide by the fundamental safety rules. Specifically, this applies to boiler users and chimney sweeps whose one duty is sweeping chimneys clear of soot. It is also required that chimney soot is rendered harmless according to the rules of environmental protection for wastes containing mercury.

### Assessment of mercury emissions into the atmosphere from the combustion of hard coal in a home heating boiler

**Fig. 6:**
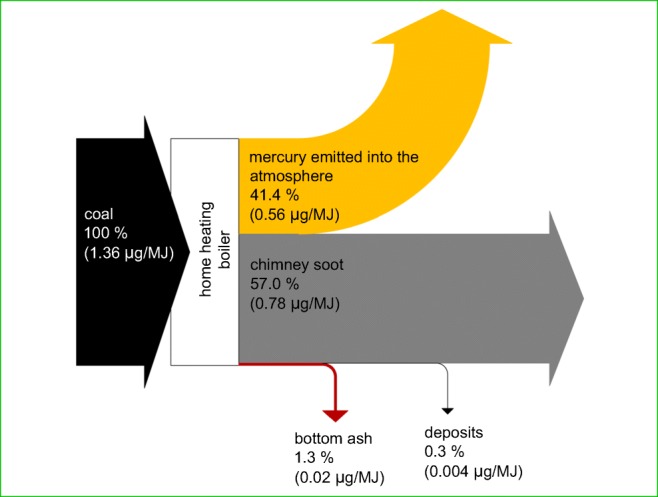
Calculation of mercury in the process of combustion of hard coal in a home heating boiler (in brackets—the quantity of mercury in relation to the calorific value of the coal analysed is provided)

On the basis of mercury content in coal, bottom ash, boiler deposits, as well as in chimney soot and their quantities, the calculation of mercury from the combustion of hard coal in a home heating boiler was done (Fig. 6). The quantity of mercury emitted into the atmosphere was defined as the closure of the calculation to 100%. From the boiler tested, 41.4% of mercury contained in the coal burned throughout the heating season was emitted into the atmosphere, 57.0% was adsorbed by chimney soot, 0.3% by boiler deposits and 1.3% passed in to bottom ash. Similar results were presented at work (Hlawiczka et al. 2003). Annual mercury emissions into the atmosphere during the heating season for the single-family house concerned were 79 mg. According to literature data (AEA 2005), in the case of the tested home heating boiler (manual fuelled), the speciation of mercury emitted into the atmosphere is as follows: 40% of elemental form (Hg^0^), 40% of oxidised form (Hg^2+^) and 20% of particulate-bound form (Hg_p_).

The factor of emission of mercury into the atmosphere was 0.56 μg/MJ. The value of this factor is nearly four times lower than the legal value in Poland, which currently is 2.0 μg/MJ (KOBiZE 2018). The obtained factor value is also lower than the value proposed in a paper (Kubica 2017) for modern class 5 boilers, which is 1.4 μg/MJ. This could be explained by the relatively low mercury content in the analysed coal, which was 1.36 μg/MJ, although as has already been mentioned, this is the content typical of coal intended for domestic users (Klojzy-Karczmarczyk and Mazurek 2013). Furthermore, it should be taken into account the fact that a significant quantity of mercury is adsorbed by chimney soot.

The obtained results should not be generalised for the whole Polish household sector. Currently, many types of boilers are commonly used by households. Various flue gas systems and different chimney construction (brick or steel) are used as well. Moreover, coals from various mines are combusted, which are characterised by different properties, among others, by various mercury content.

### Suggestions on reduction in mercury emissions by households as a result of the combustion of hard coal

**Fig. 7:**
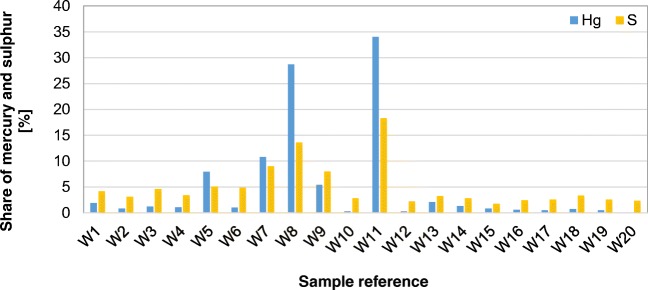
Distribution of mercury and sulphur between the coal grains analysed

One method to reduce mercury emissions from the processes of combustion of hard coal by domestic users is to previously separate the grains with pyrite overgrowths from coal. Figure 7 shows the distribution of mercury and sulphur between the coal grains analysed. The elimination of grains W8 and W11 from the combustion process would enable mercury content in the coal burned to be reduced from 32 to 15 μg/kg (a reduction by 62.7%). In the case of sulphur, the separation of pyrite overgrowths would enable 31.9% of the sulphur to be eliminated from the combustion process and its content reduced from 0.82 to 0.65% (as received basis).

The separation of grains with pyrite overgrowths from coal can be achieved using dry separators (Table 6). The results of the authors’ previous research (Dziok and Strugała 2017a) showed the possibility for reduction in mercury content by dry deshaling of coal using a vibratory air separator. For feed with a similar ash content (8.4%), the separator enabled mercury content in coal to be reduced from 79 to 37 μg/kg and high-mercury content wastes to be separated at a level of 162 μg/kg (on dry basis).

**Table 6:** Comparison of machines for dry separation of hard coal (Baic et al. 2015; Gawenda et al. 2014)

Parameter	Vibratory air separator	Optical X-ray separator
Principle of operation	The rising movement of air causes feed to be loosened, raised and suspended, the result of which is diversification by density, while the vibratory movement of a working plate causes the material to move over the working plate surface and individual products to be collected.	The separator is equipped with a system for optical and X-ray analyses, enabling the material to be separated. On the basis of analysis results, an automated decision is made whether or not to reject a given grain of the material.
Use of water	no	no
Feed particle size [mm]	0–75	8–250 (300)
Output [Mg/h]	up to 1000	up to 500
Pyrite overgrowths removal capability	Yes–Pyrite is much heavier (approx. 5 g/cm^3^) than pure coal substance (approx. 1.2 g/cm^3^), and their separation is possible.	Yes–Pyrite demonstrates a strong ability to absorb X-radiation, which enables pyrite overgrowths grains to be identified and to be rejected.
Costs compared to washing methods		
• investment costs	10–20%	60–80%
• operating costs	25–33%	10–15%

For removing grains with pyrite overgrowths from coal, an optical X-ray separator can be used as well. The machine makes use of differences in colour, particle geometry, surface structure and density. Pyrite demonstrates a strong ability to absorb X-radiation, which enables pyrite overgrowth grains to be identified without difficulty and to be separated from coal. The usefulness of this type of separator for removing mercury from coal was suggested in the results of another paper by the Authors (Dziok and Strugała 2017b).

**Fig. 8:**
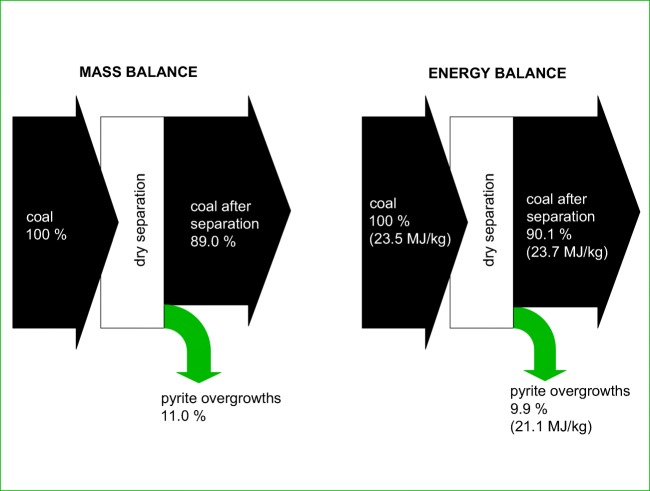
Calculation of mass energy for the process of separation of pyrite overgrowths from coal (in brackets—the calorific value of coal is provided)

It should be emphasised that the separation of pyrite overgrowths from coal results in a loss of fuel (Fig. 8). For the coal analysed, it was estimated that the loss of energy contained in the initial coal was at a level of 9.9%, and the loss of mass at a level of 11.0%. With the combustion of coal by the household sector at a level of 10 million Mg a year (GUS 2017), such a loss is significant, which makes it necessary to use the separated pyrite overgrowths for power generation. The coal grains removed are characterised by mercury content at a level of 203 μg/kg and sulphur content at a level of 2.47%, which is a certain constraint for their use.

A solution could be found in sending the separated coal grains to power plants and co-combusting them with power coal. Industrial installations are equipped with advanced flue gas treatment systems, i.e., SCR (selective catalytic reduction) or NSCR (non-catalytic selective reduction), electrostatic precipitators or bag filters, flue gas desulphurisation systems (wet or semi-dry), as well as having the capacity to use adsorbents. For Polish power plants, the effectiveness of mercury removal from flue gas achieves a level of 66% (Burmistrz et al. 2016). The cost-effectiveness of the proposed undertaking, however, requires conducting suitable research and performing appropriate economic analyses.

An additional opportunity for reducing mercury emissions by households is to use flue-gas dust-removal devices, e.g., chimney electrostatic precipitators. Their dust removal efficiency varies between 50 and 90% (Nowak and Łukasik 2017). Taking into account the fact that between 10 and 20% of mercury emitted by domestic users is the mercury adsorbed on dust (AEA Technology and NILU Poland 2005), a further 5–18% of mercury can then be removed in this manner from flue gas.

**Fig. 9:**
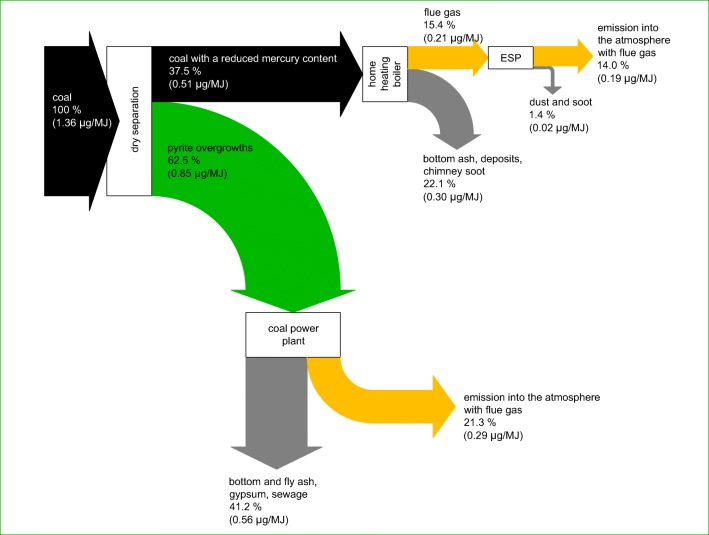
Calculation of mercury for the suggested concept of reduction in mercury emissions from the household sector in Poland (the quantity of mercury in relation to the calorific value of the coal analysed is provided in brackets)

The calculation of mercury for dry separation of coal and the use of chimney electrostatic precipitators combined is shown in Fig. 9. The concept suggested would enable mercury emissions to be significantly reduced. Mercury emissions from a heating boiler compared to its content in the initial coal would be 14.0% (0.19 μg/MJ). A further 21.3% of mercury (0.29 μg/MJ) would be emitted with flue gas in a power plant. It should, however, be emphasised that the relocation of emission from inhabited areas (cities, villages) to remote coal power plants is advantageous. This enables low emission to be reduced, which is the cause of smog during the heating season in many Polish cities and villages (GIOŚ 2017). The solution proposed would enable annual mercury emissions into the atmosphere from the domestic user sector to be reduced by 66.1% (0.397 Mg) and global mercury emissions by 0.8% (0.088 Mg). The emission factor for the tested boiler would be only 0.23 μg/MJ.

Currently, efforts with a view to reducing mercury emissions into the atmosphere are being made in Polish power plants. The implementation of methods dedicated to the removal of mercury from flue gas will bring about an increase in the reduction of mercury emissions from households for the proposed solution. The BAT provisions for large combustion plants (BAT-LCP 2017) adopted by the EU may contribute to this. Promising results in the removal of mercury from flue gas in Polish conditions were achieved when using an adsorbent on the basis of coke dust from the dry quenching of coke (Wierońska et al. 2018). It should also be emphasised that the solution proposed could also contribute to the reduction in emissions of other ecotoxic elements, e.g. arsenic, sulphur.

It should also be mentioned that there are other methods which allow for mercury removal from hard coal before its combustion, among others, the coal washing and thermal pretreatment processes. The coal washing process, like the dry separation process, allows for the removal of mercury occurring in mineral matter, mainly in pyrite. By contrast, the thermal pretreatment of coal allows for the removal of mercury occurring mainly in organic matter as well as in the inorganic constituents characterised by a low temperature of mercury release. For this reason, the effectiveness of mercury removal depends on a mode of its occurrence in coal. The effectiveness of mercury removal in the Polish industrial coal preparation plants was achieved in the range from 29 to 89% (Pyka and Wierzchowski 2016). The effectiveness of mercury removal from Polish hard coals in the pretreatment process at 300 °C conducted in a laboratory scale ranged from 45 to 70% (Dziok and Strugała 2017b). The potential of this method was confirmed by the results obtained on a pilot scale carried out by WRI (Western Research Institute). The efficiency of mercury removal at 300 °C was close to 70% (Bland et al. 2007). According to authors’ previous research (Dziok and Strugała 2017b), the combination of the coal cleaning (washing) and thermal pretreatment processes has shown the synergy effect.

## Conclusions

- It was defined that the mercury emission factor for the investigated case of a single-family house is at a level of 0.56 μg/MJ. This factor value is four times lower that the factor value presumed for the purposes of defining the mercury emissions coming from the processes of combustion of hard coal by households in Poland, which is 2.0 μg/MJ.
- It was shown that 41.4% of the mercury contained in coal burned in a home heating boiler is emitted into the atmosphere, 57.0% is adsorbed by chimney soot, 0.3% by boiler heater deposits, and 1.3% passes into bottom ash. Annual mercury emissions into the atmosphere from the combustion of hard coal throughout the heating season in the single-family house concerned were 79 mg.
- Mercury emissions can be significantly reduced by households by separating any overgrowths with pyrite from coal. To this end, dry separation devices may have application. The pyrite overgrowths separated can be burned in coal power plants, whose cleaning systems enable mercury to be efficiently removed from flue gas. Using home electrostatic precipitators provides a supplemental opportunity for reducing mercury emissions. The solution proposed would enable a reduction in annual mercury emissions into the atmosphere in Poland from the domestic user sector by 58.5% (0.351 Mg). The factor of emission of mercury into the atmosphere would be 0.23 μg/MJ.
